# QSAR based predictive modeling for anti-malarial molecules

**DOI:** 10.6026/97320630013154

**Published:** 2017-05-31

**Authors:** Deepak R. Bharti, Andrew M. Lynn

**Affiliations:** 1 School of Computational and Integrative Sciences, Jawaharlal Nehru University, New Delhi-67

**Keywords:** Malaria, apicoplast, predictive model building, R statistical package

## Abstract

Malaria is a predominant infectious disease, with a global footprint, but especially severe in developing countries in the African subcontinent. In recent years, drug-resistant malaria has become an alarming factor, and hence the requirement of new and improved drugs is more crucial than ever before. One of the promising locations for antimalarial drug target is the apicoplast, as this organelle does not occur in humans. The apicoplast is associated with many unique and essential pathways in many Apicomplexan pathogens, including Plasmodium. The use of machine learning methods is now commonly available through open source programs. In the present work, we describe a standard protocol to develop molecular descriptor based predictive models (QSAR models), which can be further utilized for the screening of large chemical libraries. This protocol is used to build models using training data sourced from apicoplast specific bioassays. Multiple model building methods are used including Generalized Linear Models (GLM), Random Forest (RF), C5.0 implementation of a decision tree, Support Vector Machines (SVM), K-Nearest Neighbour and Naive Bayes. Methods to evaluate the accuracy of the model building method are included in the protocol. For the given dataset, the C5.0, SVM and RF perform better than other methods, with comparable accuracy over the test data.

## Background

Malaria is endemic in many tropical and subtropical regions causing high mortality and morbidity. In the last 10-15 years, due to efforts of a global malaria eradication campaign, a significant fall has been observed in malaria infection cases. However, at the end of 2015, there were 212 million new cases of malaria and 429 thousand deaths have been reported across the globe. The majority of death cases have been recorded in Africa (~92 %) and the South- East Asia Region (~6%) [1].

Artemisinin derivatives are regarded as most effective drugs against malaria since the mid-1990s. In 2005, the WHO has recommended artemisinin-combination therapies (ACTs) be the first-line treatments for P. falciparum malaria worldwide [2]. The Artemisinin-derived molecules (ACTs) have a broad spectrum of activity (more than 120 targets) against many biologically important pathways of Plasmodium [3]. Despite their effectiveness, drug-resistant malaria has been emerged in many Asian and African countries in recent years [4,5, 6,7]. This scenario threatens the worldwide efforts for complete eradication of malaria and hence it is imperative to identify more drug targets as well as potent drugs to regulate the disease before current therapeutic agents lose their clinical relevance. Studies reveal that one of the most promising targets is the apicoplast due to its involvement in many essential biological pathways unique to Plasmodium [8].

An apicoplast is a non-photosynthetic vestigial plastid, bounded by four membrane layers, which occurs in almost all apicomplexan parasites. It has a 35 kb circular DNA quite similar to a cyanobacterial genome, which encodes approximately 55-60 genes of unknown functionality. However, Its presence is crucial for the cell [9]. There are various genetic and pharmacological studies, which confirm its essential role in cell survival. Genome analysis of apicoplast indicates their role in the biosynthesis of many important products including type II fatty acids, heme and ironsulphur cluster, and isoprenoid precursors [10]. The pathways related to above products are essentially similar to those of bacteria due to their endosymbiotic origin and entirely different from the pathways of the host organism. There were many antimalarial drugs proposed which targets cellular machinery (proteins/DNA) essential for cell survival ranging from replication, transcription, translation (parasite as well as apicoplast), fatty acid biosynthesis, heme, Iron-sulphur cluster and isoprenoid synthesis (exclusive to apicoplast). Earlier, targeting products of apicoplast gained popularity e.g. FASII pathway, but several genetic and pharmacological studies show evidence for the off-target activity of the inhibitor [11]. There were some successful attempts of targeting isoprenoid pathway [12] and heme biosynthesis [13], [14] already reported. Beside those anabolic pathway-based drug targets, efforts have been made to obstruct the cellular processes of apicoplast such as replication [15], transcription [16] and translation [17], as these processes are known to be quite similar to those of bacteria. Hence, antibacterial drugs are also considered as potential drugs for the malaria parasite. Recent reviews have listed various targets and related drugs [18,19,20]. A detailed view of target proteins summarizes pathways and drug candidates are listed in Table 1. In the present study we are focus on predictive model building using bioassay data causing delayed death in malaria parasites. A delayed death is the very interesting phenomena where parasites survive, infect and multiplied but progeny is unable to infect host.

With advancement in high-throughput bioassay techniques and computational resources, managing structural information along with bioactivity reading has become a well-established practice. This information can be utilized to screen large chemical libraries virtually, which reduces the cost and time for identifying potential drug-like molecules for further screening stages. One approach to applying this information is predictive model building. In recent years, numerous successful implementations of machine learning (ML) techniques are published for virtual screening of biologically active compounds [21,22, 23,24]. In the present study, we employed various state of the art machine-learning techniques to build classification models using publicly available antimalarial bioassay data with known inhibitory effect against apicoplast formation.

**Figure 1:**
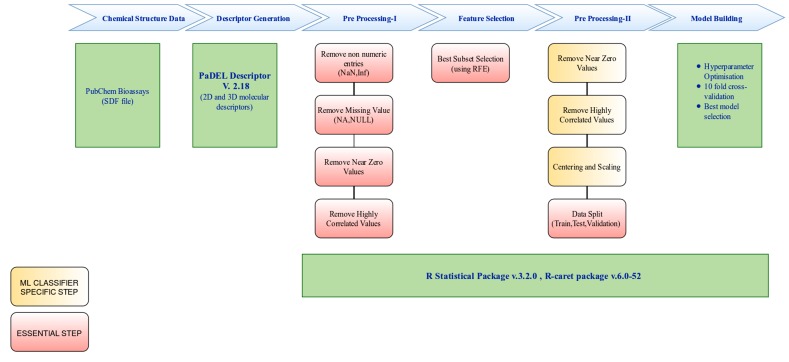
Workflow adopted for the current study. The initial dataset is in SDF format. Descriptors are calculated, and preprocessing-I is applied regardless of data and the applied Machine Learning (ML) method. The preprocessed data was subjected to Recursive Feature Elimination (RFE) based feature selection method to obtain the best feature subset for model building. The input data is prepared according to the selected feature set, and preprocessing-II was applied which solely depends on best practices suggested by caret package for the underlying ML method. The model building step includes hyper parameter optimisation, cross-validation and best model selection steps. The output is a model file which can be further used for prediction of unlabelled compound libraries. The preprocessing and model building step has been carried out by using R and the caret package.

To build a robust predictive model we define best practices for data cleaning, preprocessing, feature selection and model building, which are described in this manuscript. A schematic overview of the model building workflow can be seen in Figure 1, and is described in detail in the next section. The methods are applied on datasets to build models against targets specific to the apicoplast.

## Methodology

### Data

In the present study, we used cell-based bioassay data [AID- 504832] downloaded from PubChem [25]]. The dataset consists of 305,803 compounds including 18,126 biologically active compounds against apicoplast formation in Plasmodium falciparum. The dataset of active and inactive compounds are obtained as 2D Structure Data Format (SDF) and converted into 3D SDF file using the corina package [26].

### Descriptor Generation and Data Preparation

2D and 3D descriptors were generated for active and inactive compounds using descriptor calculation package, PaDEL v2.18 [27]. It calculates 1786 different descriptors. In our study, we calculated only 1D, 2D and 3D descriptors (without PubChem fingerprint descriptors). The redundant and missing entries have been removed from the datasets. We also excluded near zero variance and highly correlated values (>= 0.80) from the data, as they do not provide any improvement to the learning. After applying the above preprocessing step 173 predictors remained for model building. All preprocessing steps were done using R version 3.2.0 [28].

### Feature Selection

Feature selection has many important aspects including a reduction in dimensionality of data, storage requirement and learning time. We used the R-caret package for feature selection called "Recursive Feature Elimination (RFE)" [29], [30]. This method uses various functions for selecting the best feature subset from the available feature set, which is sufficient to characterize a hidden pattern from the data set responsible for defining a class. We used the random forest based function with 3-fold cross-validation to select the best feature subset. We obtained a set of 50 features, which are sufficient for the classification task.

### Classifiers

We used R-caret package for employment of various state of the art ML methods for the predictive model building including Generalized Linear Model (GLM), K-Nearest Neighbour (KNN), Support Vector Machine (SVM), Random Forest (RF), and C5.0 decision tree (C5.0). The input data set was randomly divided into training and test set with a 1:4 ratio. From the training data 25% data is kept for validation set and the remaining 75 % training data is used for model building. Each model is built using 10 fold crossvalidation (repeated 10 times) with "boot632" re-sampling method to check the robustness of the model. During cross-validation, each ML method is fine-tuned over a range of respective parameter values. The best model was selected using performance over the validation set and used for performance evaluation with test data.

### Statistical Measures for Performance Evaluation

**Figure 2:**
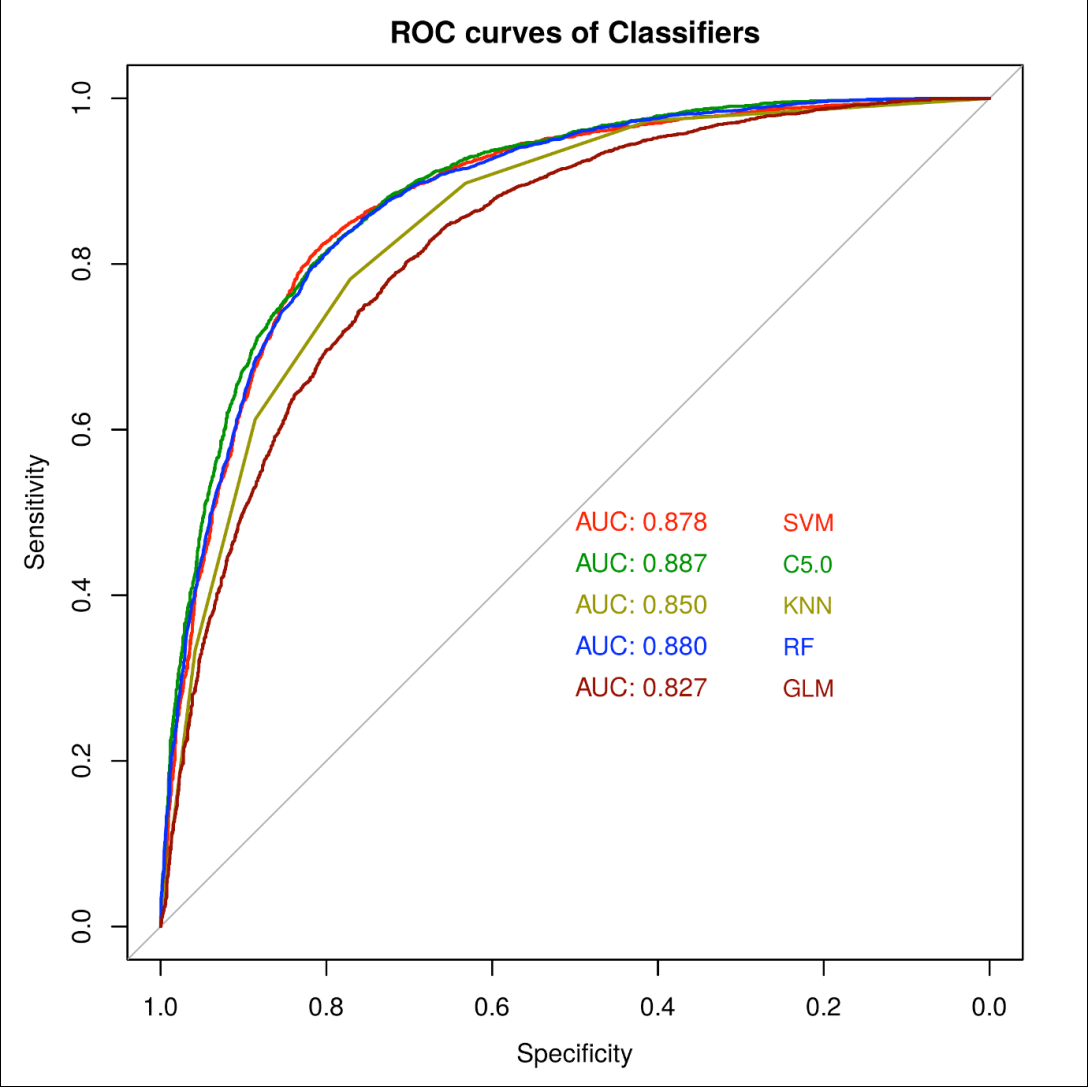
The ROC plots for different classifiers with AUC values. The higher AUC values indicates better prediction power of concerned machine learning method.

Various statistical performance measures are employed to evaluate models. Sensitivity (TP/TP+FN) measures the correctly identified positive cases while Specificity (TN/TN+FP) measures the correctly identified negative cases. Precision (TP / TP+FP) is a measure of the fraction of retrieved instances that are relevant; Accuracy (TP+TN/TP+TN+FP+FN) on the other hand measures the proximity of being true. A Receiver Operating Characteristic (ROC) is another widely used measure for classification model evaluation. The Area Under Curve (AUC) is used to evaluate the model (Figure 2). The Kappa value also provides evidence of the goodness of model. The Kappa value is a metric that compares an observed accuracy with an expected accuracy (i.e. .by random chance). Its values lie between 0-1. The higher the value, the better the model is. MCC values are indicator of quality of binary (two-class) classification model. Its value lies between -1 to 1. The positive value indicates better model.

## Results & Discussion

The datasets used in our study was a confirmatory bioassay. The initial 2D dataset (18,126 active - 98878 inactive) was subjected to 3D conversion, and then 2D and 3D molecular descriptors were calculated. There were a total of 905 descriptors computed. Some compounds, which failed to convert in 3D or to produce molecular descriptors, are discarded from the study. After descriptor calculation 18109 active and 98878 inactive compounds are retained for preprocessing-I. The first step of preprocessing-I is a removal of missing values. In the present study, we removed all columns having equal to more than 10 % missing values, which resulted in the exclusion of 20 columns. Again we removed all such rows having any missing values. The benefit of two level missing value handling is we can keep as many as samples for model building. The second preprocessing-I step is an elimination of near zero values (NZV). The descriptors having a majority of only a single value across the column does not contribute to model's prediction power while increasing the cost in terms of computational time. Hence, it is one of the best practices, applied for data cleaning. There were 420 descriptors removed during this process, and 465 descriptors were retained. We also removed highly correlated data points (cutoff >= 0.8) which resulted in the exclusion of 292 descriptors thus after preprocessing-I only 173 descriptors remained. The preprocessed data was subjected to feature selection. We implemented RFE based feature selection procedure to obtain best feature subset. The feature subset selected using 3 fold crossvalidation, over a different size of feature subset ranging from 40 to 173 with an interval of 5. The best subset obtained on feature subset of size 50 by maximum accuracy achieved. Those 50 features were utilized as input dataset features for model building.

The preprocessing-II step includes splitting of data into train and test set. Other steps are optional and depend on the recommendation for ML method under study. To address the class imbalance problem, we applied down sampling of the data (random sampling has been done to bigger class and choose samples equal to a smaller class). The validation set consist of 25 % cases of the training set and model was evaluated using 10 fold cross-validation to obtain the best robust model. The model's performance measures were checked on test data set and shown in Table 1. RF and C5.0 outperform the rest while GLM gives the poorest result. SVM (RBF) also gives an excellent result. The ROC curve analysis (Figure 2) and Cohen׳s kappa values and MCC values also strengthen the above observations. In ROC analysis C5.0, RF and SVM outperform the rest while GLM has lowest AUC value which again supports the superiority of C5.0, SVM and RF classifiers. The kappa value of C5.0, SVM and RF are also high and comparable as well. The SVM, C5.0 and RF classifiers perform almost equally well and possess almost similar performance over test set.

The QSAR model built can be used to screen the potential molecules for next phase screening. The models built in these studies will be available on request. To check the robustness of created model we additionally screen various related bioassay dataset [ID-488745, AID-488752, and AID-504848]. The results are summarized in Table 3.

The present study can be further extended. The dataset used in the study, are a set of compounds, which inhibit apicoplast formation by targeting cellular processes like replication, transcription and translation hence can be used to screen only those libraries, which possess similar targets. Second, we did not evaluate many powerful machine-learning methods like boosting, bagging, neural network and other hybrid classifiers. Hence exploring other machine learning methods with different feature selection and generation may be investigated. Third, the prediction task requires a same computational environment used for model building.

## Conclusion

In the present study, we have defined best practices for predictive model building in cheminformatics. Briefly, the initial dataset is normally present in a suitable format such as mol or sdf. If the sdf has only 2D information, it is converted to 3D information. The descriptor calculation is followed by a preprocessing step, which is split, into two tracks, the second of which is optional and depends on classifier used. The preprocessed input data is subjected to feature selection and best feature subset containing data set is used for model building purpose. The model-building step supports parallel computation, which ensures minimum time for a model generation with tuned parameters. The best model is chosen based on cross-validation results and prediction is done using best model.

The workflow is applied in the predictive model building of biologically active inhibitor molecules against apicoplast formation. Such predictive models are essentially required to facilitate rapid first level selection of potential drug-like molecules. We compared the performances of few state of the art machine learning techniques and also applied context based data pre-processing, feature selection, and Cross-validation which are known for significant influence over model performance and robustness. The under sampling of data and parallel computation results in smaller computational time with overall good results. In our study RF, C5.0 and SVM performed very well and achieved comparable predictive power. All predictive models and R-scripts are available freely on request.

## Figures and Tables

**Table 1:** Antimalarial drugs with targets

Pathway /Process	Targets	Drugs	Source(s)
Replication	GyrA, GyrB	Fluoroquinolone, Ciprofloxacin, Clindamycin, Doxycycline, Novobiocin, Coumermycin,chloroquine	[15],[18],[19],[31], [32]
Transcription	RpoB, RpoC1, RpoC2	Rifampin, Thiostrepton, Doxycycline, Tetracycline, Clindamycin	[16],[18],[33][33], [34]
Translation	Pf1F-1, 23s rRNA, GTPase, Aminoacyl tRNA - synthetase,PTC	Macrolides, Thiostrepton, Chloramphenicol, Lincosamides, Micrococcin, Mupirocin,Indolmycin	[17],[18],[20],[35], [36]
Fatty acid biosynthesis	FASII, FabH, FabI, β-ketoacyl-ACP sythetase I and II	Thiolactomycin, Cerulenin, Triclosan	[18]
Isoprenoid synthesis	DOXP reductoisomerase	Fosmidomycin	[12]
Heme Synthesis	Dehydratases	Herbicides	[13],[14]

**Table 2:** Performance of various models on train and test data sets (boot632 re-sampling, 10-fold cross validation repeated 10 times. Values are up to 2 significant points.)

Method	ROC		Accuracy		Sensitivity		Specificity		Precision	F1-score	MCC	Kappa
Train	Test	Train	Test	Train	Test	Train	Test	Test	Test	Test	Test
GLM	0.82	0.82	0.75	0.75	0.74	0.74	0.76	0.76	0.76	0.75	0.5	0.5
RF	0.92	0.88	0.87	0.8	0.86	0.79	0.88	0.82	0.82	0.8	0.61	0.61
C5.0	0.92	0.88	0.87	0.8	0.86	0.78	0.88	0.83	0.82	0.8	0.61	0.61
SVM	0.9	0.88	0.83	0.81	0.82	0.8	0.84	0.82	0.82	0.81	0.63	0.63
KNN	0.86	0.85	0.79	0.78	0.79	0.77	0.79	0.78	0.78	0.77	0.55	0.55

**Table 3:** Model performance on previously unseen data. The bioassays under study were first cross-checked for common compounds used for model building. Only previously unseen compounds are used for model performance.

	AID-488745		AID-488752		AID-504848	
Predicted	Predicted	Predicted	Predicted	Predicted	Predicted
Active	Inactive	Active	Inactive	Active	Inactive
GLM	114/154	613/800	106/134	684/883	547/966	188/223
RF	129/154	621/800	118/134	684/883	564/966	187/223
C5.0	126/154	608/800	117/134	669/883	599/966	182/223
KNN	95/154	412/800	82/134	448/883	534/966	149/223
SVM	139/154	516/800	123/134	558/883	593/966	185/223
